# Do Gender Differences in Perceived Prototypical Computer Scientists and Engineers Contribute to Gender Gaps in Computer Science and Engineering?

**DOI:** 10.1007/s11199-017-0763-x

**Published:** 2017-04-07

**Authors:** Joyce Ehrlinger, E. Ashby Plant, Marissa K. Hartwig, Jordan J. Vossen, Corey J. Columb, Lauren E. Brewer

**Affiliations:** 1 Department of Psychology, Washington State University, Pullman, WA 99163 USA; 2 Department of Psychology, Florida State University, Tallahassee, FL USA; 3 Department of Psychology, Prairie View A & M University, Prairie View, TX USA; 4 Department of Psychology, Stephen F. Austin State University, Nacogdoches, TX USA

**Keywords:** Gender gap, STEM, Social perception, Stereotyping, Self-concept, Confidence

## Abstract

Women are vastly underrepresented in the fields of computer science and engineering (CS&E). We examined whether women might view the intellectual characteristics of prototypical individuals in CS&E in more stereotype-consistent ways than men might and, consequently, show less interest in CS&E. We asked 269 U.S. college students (187, 69.5% women) to describe the prototypical computer scientist (Study 1) or engineer (Study 2) through open-ended descriptions as well as through a set of trait ratings. Participants also rated themselves on the same set of traits and rated their similarity to the prototype. Finally, participants in both studies were asked to describe their likelihood of pursuing future college courses and careers in computer science (Study 1) or engineering (Study 2). Across both studies, we found that women offered more stereotype-consistent ratings than did men of the intellectual characteristics of prototypes in CS (Study 1) and engineering (Study 2). Women also perceived themselves as less similar to the prototype than men did. Further, the observed gender differences in prototype perceptions mediated the tendency for women to report lower interest in CS&E fields relative to men. Our work highlights the importance of prototype perceptions for understanding the gender gap in CS&E and suggests avenues for interventions that may increase women’s representation in these vital fields.

**Electronic supplementary material:**

The online version of this article (doi:10.1007/s11199-017-0763-x) contains supplementary material, which is available to authorized users.

U.S. women are vastly underrepresented in the fields of computer science and engineering (CS&E). For example, only 24.7% of computer science professionals and 15.1% of engineering professionals are women, although women constitute close to half of the total workforce (U.S. Department of Labor 2016). Underrepresentation in CS&E has significant costs both in terms of valuable opportunities that women forego and critical technology design flaws stemming from a lack of diverse perspectives (Margolis and Fisher 2002; Margolis et al. 2008). Therefore, it is important to better understand women’s underrepresentation in order to devise means of facilitating greater diversity in CS&E.

A cross-disciplinary research effort has informed our understanding of why gender gaps exist in science, technology, engineering, and math (STEM). For example, gender differences in confidence (Eccles 1987; Goodman et al. 2002), belongingness (Morgan et al. 2001), and parental encouragement (Fox et al. 1983) contribute to tendencies for women to show lower interest in STEM careers compared to men. However, there is still much to learn about the predictors of underrepresentation. In particular, much of the past research on this topic has focused on the consequences of stereotypes regarding gender. Recent research suggests that it is also important to understand the content of the stereotypes that people hold regarding STEM fields. Cheryan et al. (2009) demonstrated that environmental cues suggesting that computer science was a “geeky” field led women to feel lowered belongingness and interest in computer science. Although this work highlighted the potential importance of understanding the stereotypes that exist about STEM fields, it focused on the impact of environmental cues and did not directly measure participants’ perceptions of computer science.

The present investigation builds on this work by directly measuring men’s and women’s perceptions of the intellectual characteristics (e.g., intelligent, logical) of prototypical individuals in computer science or engineering. To shed light on the gender gap in CS&E, we examined the relationship between prototype perceptions and students’ interest in these fields. Past research suggests that an important step in determining interest in many activities is for people to imagine the prototypical person who would engage in a given pursuit and to determine whether they are similar to that prototype (Niedenthal et al. 1985; Setterlund and Niedenthal 1993). For example, people are more likely to engage in smoking (Spijkerman et al. 2005) and drinking behaviors (Gerrard et al. 2002) to the degree that they report sharing important characteristics with prototypical smokers and drinkers. With respect to studying STEM, Hannover and Kessels (2004) discovered that high school students who reported feeling the most similar to prototypical science students also expressed the most liking for scientific subjects, relative to students with lesser feelings of prototype similarity. Cheryan and Plaut (2010) also found that students’ perceived similarity to computer scientists (as measured by a single question) predicted their interest in computer science over and above the impact of social identity threats and expectations of success.

The current research focuses specifically on perceptions of dissimilarity in intellectual characteristics. Past research suggests that individuals in CS&E are stereotyped as being highly intelligent and as possessing strong science-relevant characteristics (Baylor and Plant 2005; Cheryan et al. 2009; Margolis and Fisher 2002; Margolis et al. 2008). We hypothesize that men and women are likely to differ in two important respects that have relevance for perceptions of similarity to CS&E prototypes. First, we draw upon past research suggesting that women tend to report less confidence than do men in their intellectual and mathematical abilities (Eccles 1987; Eccles et al. 1999; Ehrlinger 2008; Ehrlinger and Dunning 2003). Thus, drawing upon past research, we hypothesize that women will rate their intellectual abilities less positively than will men (Hypothesis 1). To the degree that prototypical individuals in CS&E are viewed as high in ability, a tendency for women to view their own intellectual characteristics less positively than do men, by itself, would leave women feeling less similar to CS&E prototypes than would men.

## Gender Differences in Prototype Perceptions

In addition to any gender difference in self-views, we suggest that men and women are likely to differ in their perceptions of the prototypical person who pursues computer science or engineering. Past research on prototype matching has primarily focused on how people’s self-views differ from a commonly held view of the prototype (e.g., Niedenthal et al. 1985; Setterlund and Niedenthal 1993). However, perceptions of prototypes are likely to stem from unique information and experiences as well as from information and experiences that are shared across people. No doubt, people’s perceptions of prototypical individuals in CS&E stem, in part, from well-known media portrayals of people who enjoy CS&E (e.g., computer programmers and engineers in popular TV shows and movies; Mastro et al. 2005) and from cultural stereotypes regarding people who pursue CS&E. In addition, perceptions of prototypes are likely to be shaped by more unique experiences, including firsthand experiences with computers or engineering in the classroom and at home as well as one’s knowledge of and experiences with friends or family members in CS&E.

We propose that there might be important differences between men’s and women’s perceptions of CS&E prototypes because men and women often differ in their exposure to and experiences with CS&E. On average, men tend to have more exposure to CS&E throughout their schooling and the exposure that they do receive to these fields is often in contexts that are qualitatively more positive than is often the case for women’s exposures to CS&E (Jacobs et al. 2005; Nelson and Cooper 1997; Tiedemann 2002; for review, see Margolis and Fisher 2002). By virtue of such differences then, we propose that women likely hold more exaggerated and stereotype-consistent views of the characteristics of CS&E prototypes than do men (Hypothesis 2).

This difference in perceptions of the prototype is expected to exacerbate women’s feelings of dissimilarity to CS&E prototypes and to undermine their interest in these fields. Thus, we expected that women will give less positive ratings of overall similarity between the self and CS&E prototype than men will, which would negatively predict women’s interest in future courses and careers related to CS&E (Hypothesis 3). More importantly, we expected that women will give higher ratings of the intellectual characteristics of CS&E prototypes that, in turn, will mediate the gender difference in CS&E, independent of gender differences in self-views (Hypothesis 4).

## The Present Studies

We designed two studies to directly examine male and female participants’ perceptions of prototypical individuals in CS&E. Participants were asked to rate the prototypical computer scientist (Study 1) or engineer (Study 2) on a series of traits and abilities. To determine the degree to which participants viewed their own characteristics in ways similar to the characteristics of the prototypical person in CS&E, we asked them to rate themselves on the same set of traits and abilities. Participants also provided an overall similarity rating comparing themselves with the prototype they had imagined. Finally, participants reported their level of interest in pursuing future courses and careers related to computer science or engineering and, in Study 2, they additionally reported their level of prior exposure or contact with engineering students and professionals. These additional measures in Study 2 were designed to explore possible gender differences in exposure to individuals in engineering and whether lower levels of exposure might contribute to women’s more extreme and stereotype-consistent views of the prototype. We predicted women would report lower exposure to individuals in engineering than would men (Hypothesis 5). Furthermore, we expected that a gender difference in exposure to CS&E individuals would mediate the proposed gender difference in perceptions of CS&E prototypes (Hypothesis 6).

## Study 1

In Study 1, we asked male and female college students to rate the degree to which the prototypical computer scientist possesses a series of traits and abilities, including CS-relevant intellectual characteristics. We counterbalanced ratings of the prototypical computer scientist with a second set of items in which we asked participants to rate themselves on the same set of traits and abilities. We also asked participants to provide an overall similarity rating comparing themselves with the prototype they had imagined. Finally, participants were asked to report their interest in future CS courses and careers.

We predicted that, relative to men, women would offer more extreme, stereotype-consistent ratings of intellectual characteristics for the prototypical computer scientist. A simple tendency for women to rate the CS prototype higher than men could stem from a potential gender difference in the acquiescence bias, or the tendency to provide more positive ratings on scales, independent of the content of individual items (Bentler et al. 1971). To our knowledge, however, there exists no evidence of gender differences in the acquiescence bias. More importantly, we also predicted both that women would rate their own intellectual characteristics *less* positively than would men and that women would report *less* interest in CS than would men. Further, we expected that women’s ratings of overall similarity between the self and prototype would be lower than men’s and would predict their lower interest in CS. Finally, we expected that a tendency for women to view the intellectual characteristics of CS prototypes more positively than men would lead women to report lower interest in CS courses and careers relative to men, independent of the impact of self-views on interest.

### Method

#### Participants

Participants were 96 undergraduate students (71.9%, or 69, women) at a private university in the northern United States who participated in exchange for extra credit in a psychology course. Due to experimenter error, detailed demographic data were not collected. Consistent with university demographics, the sample was likely to be predominantly White and non-Hispanic and of traditional college age.

#### Materials and Procedure

To encourage reflection on the prototype, we asked participants to describe the prototypical computer scientist in a pencil-and-paper questionnaire:

Please take a moment to think about the features that you think characterize your average computer scientist. Once you have this picture in mind, write a few sentences describing your picture of the average computer scientist. For example, what does this person look like, what personality traits might he or she have, what types of activities would he or she enjoy, etc.

The descriptions provided were, unfortunately, too short and variable across participants to allow for meaningful content analysis. Participants were then asked to complete a one-item similarity measure that asked participants to rate “to what extent is the description that you just wrote of a computer scientist similar to you?” on a 9-point scale from 1 *(not at all similar)* to 9 *(extremely similar)*. Next, participants were asked to “Please rate the extent to which you think the following qualities characterize the average computer scientist.” They completed this task by rating the extent to which the prototypical computer scientist possessed each of a set of 13 traits and abilities on a set of 9-point scales ranging from 1 *(not at all)* to 9 *(extremely)*. Participants also rated the degree to which they possessed each of the 13 traits and abilities, using a parallel set of 9-point rating scales.

Self-ratings and prototype ratings were counterbalanced for order across participants, and all variables presented here were tested for differences based on whether the participant completed the self-ratings or prototype ratings first. No order effects were found, and, therefore, order will not be discussed further. Of particular interest was participants’ self and prototype ratings for three intellectual characteristics relevant to CS: intelligent, logical, and mathematical. The remaining 10 traits and ability ratings served as fillers to reduce participants’ focus on the target items and were less relevant to intellectual stereotypes for CS (i.e., cynical, artistic, energetic, insecure, clumsy, social, introverted, creative, studious, athletic). Finally, in order to assess interest in CS, participants were asked to rate how likely they were to “take computer science courses in future semesters” at their university and how likely they were to “choose a career that requires knowledge of computer science” on 9-point scales ranging from 1 *(not at all likely)* to 9 *(extremely likely*).

### Results and Discussion

#### Perceived Characteristics of the Self and of CS Prototypes

To examine participants’ perceptions of their own and the prototypical computer scientist’s characteristics, we began by conducting two principal components analyses using direct oblimin rotation—one conducted on the set of 13 trait self-ratings and one on the parallel set of 13 ratings of the prototypical computer scientist—in order to identify whether similar factors emerged across the self and prototype ratings representing the intellectual traits most relevant to our hypotheses.

We found that the factors that emerged in participants’ ratings of the prototypical computer scientist differed substantially from the factors that emerged when participants rated their own characteristics. As can be seen in Table 1, five factors emerged with eigenvalues greater than one for both the self and the prototype ratings. However, the items that define these latent constructs differed between ratings of the self and those of the prototypical computer scientist. For example, participants’ ratings of athleticism, artistry, and creativity loaded onto the same factor when participants rated the CS prototype but, when completing self-ratings, these items loaded onto three separate factors. Even more dissimilar factors emerged when responses from male and female participants were looked at separately. (See Tables 1s and 2s of the Online Supplement for results of factor analyses separated by gender.) There was, however, some consistency with respect to a factor representing the intellectual traits across self and CS prototype ratings, with three items (intelligent, mathematical, and logical) falling into the same factor. Note, however, that even this factor did not align perfectly because participants’ ratings of their own intellectual traits loaded on the same factor as their “energetic” and “athletic” self-ratings, whereas the same was not the case for participants’ ratings of the CS prototype.

**Table 1:** Factors for ratings of the computer science prototype and self, Study 1

	Factors rating the computer science prototype					Factors from self ratings				
Items	1	2	3	4	5	1	2	3	4	5
Athletic	.48				-.44		.65			
Artistic	.78									-.91
Creative	.82									-.91
Energetic	.77					.62	.48			
Studious	-.45						.72			
Intelligent		.52							-.42	
Logical		.83					.42	-.52		
Mathematical		.83				-.47	.47			
Introverted			.82			-.83				
Social			-.76			.81				
Cynical				.80				-.61		
Insecure				.71					.92	
Clumsy					.90			.76		
% of variance explained	25.48	12.14	10.37	8.88	7.71	21.25	16.24	11.40	8.91	8.60

Results from direct oblimin rotations performed separately on prototype and self-ratings. All loadings greater than .40 are shown

In retrospect, it is perhaps not surprising that people view themselves in broad terms while viewing prototypical others, like all outgroup members, in more narrow ways (Park and Rothbart 1982). This finding, however, made it impossible to create a composite variable for intellectual traits drawn from a single factor that is identical in both self-ratings and ratings of the prototypical computer scientist. Because our primary hypotheses were focused on perceptions of the intellectual characteristics of the prototype (rather than self-views), we created composite variables based on the intellectual factor that emerged from ratings of the prototype. For this reason, we expected higher reliability among the ratings that made up this composite for the prototype than for self.

We averaged participants’ ratings of the degree to which the CS prototype was viewed as intelligent, mathematical, and logical to create a composite variable representing perceived intellectual characteristics of the CS prototype. The reliability of these ratings for the prototypical computer scientist was acceptable (α = .63). We computed a parallel composite measure indicating the degree to which participants rated their own intellectual characteristics positively by averaging self-ratings on the same three traits. Reliability for participants’ self-ratings for the same characteristics was low (α = .45), for the reasons discussed. Despite the lower reliability in our composite for self-ratings, we thought it important to include this composite measure in the analyses as an important comparison point for participants’ ratings of the prototype.

Next, we examined the prediction that women would rate their own intellectual characteristics *lower* than would men but the prototypical computer scientist’s intellectual characteristics *higher* than would men. We conducted a 2 (male vs. female) × 2 (self vs. prototype rating) repeated measures ANOVA to compare men’s and women’s ratings of the self and the prototype. This analysis revealed a significant main effect of rating target, indicating that participants offered higher ratings of intellectual characteristics for the prototypical computer scientist than for themselves, *F*(1,94) = 171.05, *p* < .001, ηp^2^ = .65 (see Table 2). As predicted, this main effect was qualified by an interaction between rating target and participants’ gender, *F*(1,94) = 25.39, *p* < .001, ηp ^2^ = .21. Consistent with past research, female participants offered *lower* ratings of their CS-relevant abilities than did men, *F*(1,94) = 16.35, *p* < .001, ηp ^2^ = .15. Importantly, female participants also offered *higher* ratings of the prototypical computer scientist’s ability than did their male counterparts, *F*(1,94) = 5.34, *p* = .023, ηp ^2^ = .05 (see Table 2 for means). Thus, men and women differ not only in their self-views but also in their perceptions of prototypical individuals in computer science.

**Table 2:** Gender differences in intellectual ratings of the self and the computer science prototype, Study 1

Rating	Total *M* (*SD*)	Women *M* (*SD*)	Men *M* (*SD*)	Gender difference
Self	6.17_a_ (1.18)	5.88_a_ (1.13)	6.89_a_ (1.00)	-1.01
CS prototype	8.21_b_ (.67)	8.30_a_ (.67)	7.96_b_ (.61)	.34
Target difference	-2.04	-2.42	-1.07	

Means with differing subscripts within the total column and between women and men are significantly different, *p* < .05

#### Perceived Overall Similarity of the Self and Prototype

We conducted a *t*-test to examine whether men and women differed in their perceptions of similarity to the CS prototype. This analysis revealed, as predicted, that female participants (*M* = 2.95, *SD* = 1.72) rated themselves as less similar to the prototype than did men (*M* = 3.80, *SD* = 1.94), *t*(94) = 2.10, *p* = .039, *d* = .46.

#### Interest in CS

We next calculated a composite variable representing interest in CS (where higher scores represented stronger interest) by averaging participants’ ratings of (a) their likelihood of taking CS classes in the future and (b) their likelihood of choosing a career that requires knowledge of CS (α = .88). We then conducted a *t*-test to explore our prediction that women would report lower interest in future CS courses and careers compared to men. Indeed, female participants (*M* = 2.60, *SD* = 1.93) reported substantially lower interest in future CS courses and careers than did their male counterparts (*M* = 4.33, *SD* = 3.06), *t*(94) = 3.32, *p* = .001, *d* = .68.

To explore the role of prototype perceptions in producing the observed gender difference in interest, we conducted two mediation analyses. We first examined whether the relationship between gender and interest was mediated by overall gender differences in feelings of similarity to the CS prototype. A bias-corrected bootstrapping PROCESS Model 4 analysis (Hayes 2013) with 10,000 samples revealed the predicted indirect effect of gender on interest through participants’ perceived overall similarity between the self and the prototype (*ab* = .53, *SE* = .30, 95% CI [.04, 1.22]). This analysis suggested that, relative to men, women offered lower ratings of similarity to the prototype (*a* = .85, *SE* = .40, 95% CI [.04, 1.65]), which, in turn, predicted lower interest in CS courses and careers (*b* = .62, *SE* = .12, 95% CI [.39, .86]). The remaining direct effect in this model was significant (*c’* = 1.20, *SE* = .47, 95% CI [.27, 2.14]).

We conducted a second PROCESS Model 4 analysis (Hayes 2013) to examine the more novel and specific hypothesis that the effect of gender on interest was mediated by gender differences in perceptions of CS prototypes, controlling for self-views. This analysis indicated the proposed indirect effect was significant (*ab* = .28, *SE* = .18, 95% CI [.02, .76]), suggesting that intellectual perceptions of the CS prototype (and not just intellectual perceptions of the self) mediated the relationship between gender and interest in CS. Importantly, this analysis suggests that, above and beyond any effect of gender differences in participants’ self-views, women’s perception of the intellectual characteristics of CS prototypes as more positive and extreme, relative to men’s, contributes to the gender gap in CS interest.

### Summary

In Study 1, we found that women offered less positive estimates of their own intellectual abilities than did male participants, but more positive (i.e., stereotype-consistent) estimates of the intellectual abilities of the prototypical computer scientist than did men. We also tested our prediction that women’s more extreme perceptions of prototypical individuals in CS relative to those of men would contribute to a gender difference in CS interest. Indeed, a mediational analysis revealed an indirect effect whereby women reported feeling less similar to CS prototypes than did men which, in turn, predicted lower reported interest in future CS courses and careers compared to men. Further, women reported more positive and stereotype-based perceptions of the intellectual characteristics of CS prototypes compared to men, a difference that mediated the gender difference in CS interest.

## Study 2

Study 2 was designed as a replication and extension of our first study. In Study 2, we examined whether men and women differed in their perceptions of prototypes in a field related to computer science and characterized by similar stereotypes: engineering. We also went beyond the previous study by having participants rate their level of exposure to the field of engineering through the number of engineering students or professionals they knew personally and their amount of personal contact with them. These additional items allowed us to evaluate whether lower exposure to the field of engineering can help to explain why women’s perceptions of engineering prototypes are more extreme than men’s.

### Method

#### Participants

The sample was made up of 173 undergraduate students (68.2%, or 118, women, 100% White, and 12.7%, or 22, Hispanic or Latino) at a large U.S. Southeastern public university who participated in exchange for credit toward their undergraduate psychology courses. Participants ranged in age from 17 to 32 years-old (*M* = 18.95, *SD* = 1.76, *Mdn* = 18), and 115 (66.5%) were first-year students, 27 (15.6%) were sophomores, 17 (9.8%) were juniors, and 14 (8.1%) were seniors. There were no ethnicity or class year differences between women and men in our sample. Age differed slightly between women (*M* = 18.7 years, *SD* = 1.2) and men (*M* = 19.5 years, *SD* = 2.5), *t*(171) = 2.15, *p* = .035, *d* = .39, but because age was not correlated with any of our dependent measures (see Table 3s of the Online Supplement for these correlations) and was not theoretically expected to affect the participants’ responses, we do not discuss age further.

#### Materials and Procedure

The pencil-and-paper materials and the procedure in Study 2 were identical to those of Study 1 except that participants were asked about prototypes, courses, and careers in engineering rather than computer science. Similar to Study 1, participants were first asked to reflect on the prototypical engineer by writing a few sentences describing the physical appearance, personality, or other perceived features of the average engineer. They next rated the degree to which they would describe themselves as “similar to the prototypical engineer” they had just described on a 9-point scale from 1 *(not at all similar)* to 9 *(extremely similar*). Next, participants completed two sets of trait ratings to describe the degree to which they and, separately, the prototypical engineer possessed each of nine traits and abilities taken from Study 1. As in the previous study, each rating was made on a 9-point scale from 1 *(not at all)* to 9 *(very much*). As in Study 1, the list of traits included three dimensions relevant to intellectual stereotypes for engineering (intelligent, logical, and mathematical). The remaining items served as fillers to reduce participants’ focus on these target items. We used a smaller set of filler items in Study 2, relative to the first study, to decrease the time taken to complete the set of ratings and to better explore a possible social factor. Specifically, we retained five items from Study 1 (cynical, clumsy, social, introverted, athletic) and added one additional social item (shy). Next, using 9-point scales parallel to those used in Study 1, participants rated the likelihood that they would take classes in which they would “learn engineering concepts and principles” and their likelihood of choosing “a career for which understanding engineering principles will be required.”

In this second study, participants were asked two new questions designed to measure their exposure to individuals involved in engineering. Specifically, participants were asked to select one of five response categories that best represented the number of engineering students or professionals they knew personally. Participants could indicate that they knew no students and professionals (*category 1*), 1–2 (*category 2*), 3–4 (*category 3*), 5–8 (*category 4*) or 9 + (*category 5*) students or professionals. Using a parallel set of response categories, participants were also asked: “In a typical month, during how many days do you come in contact with engineering students or professionals?” They chose the response category that best fit the frequency with which they came in contact with engineering individuals from the following options: 1 (*zero*), 2 (*1 to 2*), 3 (*3 to 4*), 4 (*5 to 8*) or 5 (*9+*) days in a typical month.

### Results and Discussion

#### Perceived Characteristics of the Self and of Engineering Prototypes

As in the previous study, we conducted two principal components analyses using direct oblimin rotation of participants’ prototype ratings and, separately, self-ratings to examine whether a common factor representing intellectual characteristics emerged across the two sets of ratings. For both the self and the prototype ratings, three factors emerged with eigenvalues greater than 1.0; however, the items that defined these factors differed across self-ratings and prototype ratings (see Table 3). That said, a common factor emerged for both self and prototype ratings representing three intellectual traits (i.e., intelligent, mathematical, and logical). This factor was identical to that identified in Study 1 for the CS prototype ratings and overlapped with the similar factor in the Study 1 self-ratings. As in the earlier study, the factors that emerged for the filler items differed in content across the prototype and self-ratings. (See Tables 4s and 5s of the Online Supplement for results of separate factor analyses for women and men, respectively.)

**Table 3:** Factors for ratings of the engineering prototype and self, Study 2

	Factors rating the engineering prototype			Factors from self ratings		
Items	1	2	3	1	2	3
Shy	.80			.76		
Social	-.87			-.81		
Athletic	-.82					-.67
Intelligent		.82			.77	
Mathematical		.85			.56	
Logical		.78			.72	
Introverted			.50	.77		
Cynical			.94	.44		
Clumsy						.84
% of variance explained	29.78	23.82	11.55	24.49	17.98	13.26

Results from direct oblimin rotations performed separately on prototype and self-ratings. All loadings greater than .40 are shown

As in the previous study, our primary hypotheses emphasized perceptions of the prototype, and therefore we prioritized the intellectual factor that arose from ratings of the prototype. (See Table 6s of the Online Supplement for a comparison of women’s and men’s prototype ratings for each trait across both studies.) As such, we anticipated higher reliability for composites of prototype ratings than for self-ratings. We computed two composite variables representing the intellectual characteristics of the engineering prototype (α = .77) and, separately, of the self (α = .42), by averaging participants’ ratings for intelligent, logical, and mathematical.

As in the first study, we predicted that female participants would rate the prototypical engineer *higher* in intellectual characteristics than would male participants, but rate their own intellectual characteristics *lower* than male participants would. A 2 (male vs. female) × 2 (self vs. prototype rating) repeated measures ANOVA revealed a significant main effect of rating target, indicating that participants offered higher ratings of intellectual characteristics for the prototypical engineer than for the self, *F*(1171) = 421.56, *p* < .001, ηp^2^ = .71 (see Table 4). This effect was qualified by the predicted interaction between participants’ gender and the rating target, *F*(1171) = 15.45, *p* < .001, ηp ^2^ = .08. Post hoc analyses revealed that female participants rated their own intellectual characteristics somewhat (but not significantly) less positively than did male participants, *F*(1171) = 3.29, *p* = .071, ηp ^2^ = .02, but the prototypical engineer’s characteristics significantly more positively than did male participants, *F*(1171) = 14.48, *p* < .001, ηp ^2^ = .08 (see Table 4). Together with Study 1, this finding suggests that women perceive the intellectual characteristics of CS and engineering prototypes in more positive and, thus, stereotype-consistent ways than their male peers do.

**Table 4:** Gender differences in intellectual ratings of the self and the engineering prototype, Study 2

Rating	Total *M* (*SD*)	Women *M* (*SD*)	Men *M* (*SD*)	Gender difference
Self	6.03_a_ (1.14)	5.92_a_ (1.18)	6.25_a_ (1.04)	-0.33
E prototype	8.36_b_ (.83)	8.52_a_ (.59)	8.02_b_ (1.14)	.50
Target difference	-2.33	-2.60	-1.77	

Means with differing subscripts within the total column and between women and men are significantly different, *p* < .05

#### Perceived Overall Similarity of the Self and Prototype

We next examined the effect of gender on participants’ reported similarity to the prototype. Female participants (*M* = 3.20, *SD* = 1.99) viewed themselves as less similar to the prototype than men did (*M* = 3.96, *SD* = 2.10), *t*(171) = 2.30, *p* = .022, *d* = .37.

#### Interest in Engineering

We created a composite measure of participants’ interest in engineering (where higher scores represented stronger interest) by averaging their ratings of (a) their likelihood of taking future courses in engineering and (b) their likelihood of choosing a career in engineering (α = .89). We then conducted a t-test to examine whether rated interest differed by participants’ gender. Consistent with our predictions and with prior research, female participants (*M* = 2.26, *SD* = 1.59) offered lower ratings of interest in future engineering courses and careers than did male participants (*M* = 3.19, *SD* = 1.75), *t*(171) = 3.47, *p* = .001, *d* = .56.

We conducted a set of two mediation analyses to explore the role of prototype perceptions and perceived similarity to those prototypes in the gender difference in engineering interest. We conducted a PROCESS Model 4 analysis (Hayes 2013) to examine whether the observed gender gap in engineering interest was mediated by a gender difference in feelings of similarity to the engineering prototype. As predicted, this model revealed a significant indirect effect of gender on interest through perceived similarity to the prototype (*ab* = .24, *SE* = .12, 95% CI [.05, .51]). Relative to men, women offered lower ratings of similarity to the engineering prototype (*a* = .76, *SE* = .33, 95% CI [.11, 1.41]), which, in turn, predicted lower interest in engineering courses and careers (*b* = .31, *SE* = .06, 95% CI [.20, .42]). The remaining direct effect in this model was not significant (*c’* = .69, *SE* = .25, 95% CI [.20, 1.20]).

Next we conducted a second PROCESS Model 4 analysis (Hayes 2013) to examine the specific mediational role that gender differences in perceptions of the engineering prototype played in predicting women’s relative lack of interest in engineering, controlling for any effect of participants’ self-views. This analysis indicated a significant indirect effect (*ab* = .20, *SE* = .09, 95% CI [.06, .41]) whereby women reported more positive and, therefore, stereotype-consistent perceptions of the intellectual characteristics of the engineering prototype and, in turn, reported less interest in future engineering courses and careers.

#### Exposure to Engineering

Finally, we examined male and female participants’ self-reported exposure to engineering by creating a composite measure of participants’ exposure to engineering (where higher scores represented more self-reported exposure) by averaging their responses regarding (a) the number of engineering students or professionals they knew personally and (b) how many days during a typical month they came into contact with engineering students or professionals (α = .66). Participants’ composite scores on this exposure measure ranged from category 1 (*zero*) to category 5 (*9*+) individuals known personally or days of exposure in a typical month. The average response category was 2.46 (*SD* = 1.03), which falls in between the categories representing “1 to 2″ and “3 to 4″ individuals known personally/days of exposure in a typical month. We conducted a *t*-test to examine whether exposure differed by participants’ gender. This test revealed, however, that female participants (*M* = 2.41, *SD* = 1.04) did not significantly differ from male participants (*M* = 2.56, *SD* = .99) in their self-reported exposure to engineers, *t*(171) = .91, *p* = .36, *d* = .15.

Although women’s self-reported exposure to engineers was not significantly lower than men’s, we nonetheless evaluated the possibility that exposure mediated the relationship between gender and intellectual perceptions of the engineering prototype. A PROCESS Model 4 (Hayes 2013) analysis provided a 95% confidence interval for the proposed indirect effect which included zero (*ab* = .01, *SE* = .01, 95% CI [−.01, .06]), indicating that self-reported exposure to engineering did not explain why women rated the intellectual characteristics of engineering prototypes as more extreme in this study. Consistent with this conclusion, we also note that exposure to engineering did not correlate significantly with intellectual perceptions of the prototype (*r* = .02, *p* = .77), intellectual perceptions of the self (*r* = .10, *p* = .17), overall similarity ratings (*r* = .11, *p* = .14), or interest in engineering (*r* = .05, *p* = .52).

### Summary

Study 2 replicated the results of Study 1 and extended them to the domain of engineering. Female participants in Study 2 evaluated the intellectual characteristics of prototypical engineers as more extreme and stereotype-consistent than did male participants and reported less interest in future engineering courses and careers. As with perceptions of CS prototypes in the first study, mediational analyses suggested that both women’s tendency to view engineering prototypes as more extreme and women’s tendency to see themselves as less similar to the prototype compared to men, contributes to women’s relative lack of interest in future engineering courses and careers. Our measure of gender differences in exposure to engineering did not predict prototype perceptions or interest.

## General Discussion

We examined male and female college students’ perceptions of prototypical people in CS&E across two studies and found support for our first four hypotheses. We replicated past research in finding that women reported less confidence in their own intellectual characteristics and less interest in CS&E, relative to men (Hypothesis 1). We also demonstrated that women reported more extreme and, hence, stereotype-consistent perceptions of prototypical individuals in CS (Study 1) and engineering (Study 2) than men did (Hypothesis 2). In both studies, women perceived themselves to be less similar to the prototype than men did, and these perceptions of dissimilarity contributed to gender differences in interest in CS&E (Hypothesis 3). Furthermore, we demonstrated that the extreme perceptions of CS&E prototypes undermined women’s interest in these fields, even when controlling for the impact of self-views on interest (Hypothesis 4).

### Limitations and Future Research Directions

The present research suggests that women’s lower interest in CS&E is partly attributable to prototype perceptions that are more extreme and stereotype-consistent than those of men. But the source of gender differences in perceptions of CS&E prototypes requires further research. In Study 2, we investigated the possibility that the difference in perceptions of prototypes stems from differences in the amount of exposure that young men and women typically have to individuals in CS&E. We found, contrary to previous research, that men and women did not differ on our measure of self-reported exposure to engineering students and professionals (Hypothesis 5 unsupported). Similarly, gender differences in exposure did not explain differences in prototype perceptions (Hypothesis 6 unsupported). This finding could be interpreted as evidence against the existence of gender differences in exposure to CS&E but we suspect such a conclusion would be premature on the basis of our somewhat simplistic and self-report measure of CS&E exposure. Future research might incorporate more detailed measures of the frequency of contact with CS&E individuals (as well as CS&E content) to test whether such differences still exist today.

Further research might also measure whether gender differences in the *quality* of the exposure to CS&E might lead to differences in perceived CS&E prototypes even if the quantity of CS&E exposure was held constant across genders. For example, it might be that men’s exposure to CS&E is qualitatively more positive and characterized by acceptance and encouragement than is women’s exposure and, for this reason, men and women differ in their perception of CS&E prototypes. Consistent with this argument, past research has shown that girls receive less parental encouragement with respect to STEM fields than do boys (Fox et al. 1983). Thus, the possibility that women and men receive different messaging about CS&E fields—and that this biased messaging may produce and maintain their different perceptions of CS&E prototypes—merits further investigation.

The mechanism by which gender differences in prototype perceptions undermine interest in CS&E also requires further investigation. Women’s perceptions of CS&E prototypes as extreme and stereotype-based, relative to men’s perceptions, might suggest that women suffer from a pluralistic ignorance in which they believe, upon encountering struggles that many students face when studying challenging material, that they alone lack the intellectual skills to succeed in CS&E. Specifically, pluralistic ignorance may lead female students to draw upon their prototype perceptions and conclude that their (often male) peers who pursue CS&E better match the prototype than themselves. As a result, they may fail to seek support from peers who are facing the same challenges. This sort of pluralistic ignorance may actually perpetuate stereotype-based views of CS&E prototypes. Because it is rare for people to advertise qualities about themselves that, they believe, will impede fitting in with their peers (Noelle-Neumann 1984), students may hide their feelings about CS&E to avoid being negatively judged, ridiculed, or socially rejected. In so doing, they may miss opportunities to dispel their mistaken perceptions by bonding with others who have similar experiences and do not possess the qualities that characterize CS&E stereotypes.

Thus, perceptions of similarities (or lack thereof) between the self and the prototype may serve as cues regarding not only the degree to which one will excel in CS&E, but also the extent to which one will feel accepted in CS&E, both of which likely feed into one’s interest in CS&E. Future research should examine whether feelings of dissimilarity to CS&E prototypes lead to lower feelings of belongingness for women than for men. Past research suggests that concerns about belongingness have negative implications for interest, motivation, and achievement in general (Ryan and Deci 2000). These concerns are also important predictors of motivation and interest in academic STEM contexts (Stake and Mares 2001; Stake and Nickens 2005; Walton and Cohen 2007). For example, women hold lower expectations of belongingness in science than men do, and these feelings predict women’s lower interest in pursuing careers in science (Morgan et al. 2001).

One determinant of whether one expects to feel accepted is the degree to which one is similar to others in the group (Aronson and Worchel 1966; Lord and Saenz 1985). Thus, it seems that people perceive, at least at some level, that having similar qualities to others might have consequences for belonging. Further, people report greater feelings of belongingness in a group to the degree that the other members of the group are similar to them. For example, women anticipate feeling greater belongingness in groups with an equal number of men and women than groups with fewer women than men (Murphy et al. 2007). It would be interesting in subsequent work to explore whether the influence of prototype perceptions on interest is due to the implications of prototype perceptions for anticipated belonging.

### Practice Implications

Our current findings regarding the importance of prototype perceptions provide valuable insights into potential interventions to increase diversity in CS&E. It is worth first noting that there are important factors that make both self-views and perceptions of prototypical others resistant to change. However, researchers have also identified effective strategies for bringing about changes in both self and prototype perceptions. For example, self-views are resistant to change because people interpret new information and feedback in ways that are consistent with pre-existing views of the self (Swann 1983, 1987). Indeed, this self-verification motive often leads people to reject positive information about the self that is inconsistent with their pre-existing self-views (Kille et al. 2017; Swann and Read 1981) and to seek out feedback likely to confirm already held perspectives of the self (McNulty and Swann 1994; Swann et al. 1989). It is also challenging to improve the accuracy of people’s self-views because people’s behavior is driven by powerful motives to think positively about the self and to verify their existing self-views more so than by motives to hold accurate views of the self (Sedikides 1993). That said, an important goal of many clinical psychologists and researchers has been to identify effective means to change at least some categories of self-views (e.g., overly negative self-views; narcissistic views of the self). Thus, future intervention research could draw upon clinical psychology strategies with proven efficacy to encourage women to view their own intellectual characteristics in a more positive light (and/or, perhaps, to encourage more accuracy in men’s views of their own intellectual characteristics).

Women’s interest in CS&E might be encouraged through interventions that combat more extreme or stereotype-based perceptions of CS&E either instead of, or in addition to, trying to change women’s self-views. Certainly there also exist powerful factors that make stereotype-based views resistant to change. For example, there is ample evidence that stereotypes influence people’s initial judgments in uncontrollable, automatic ways (Devine 1989). Further, people tend to seek out information that confirms their pre-existing stereotypes (Crocker 1981; Klayman and Ha 1987). They also explain away potential evidence that might disconfirm stereotype-based views, such as extreme prototype perceptions, as either non-diagnostic (Crocker et al. 1983; Heilman and Stopeck 1985) or as representing atypical sub-groups not relevant to the pre-existing stereotype (Rothbart and John 1985; Weber and Crocker 1983).

That said, researchers have also discovered effective means of reducing stereotype-based attitudes and judgments. Most relevant to the current investigation, past research has shown that exposure to positive role models can be an important factor for increasing women’s interest in STEM (Good et al. 2010; Young et al. 2013). Women show increases in CS&E interest after interacting with an exemplar designed to counter stereotype-based views of CS&E. For example, participants engaged in a brief computer task that included interacting with a female, socially skilled avatar described as an engineer. Relative to control participants, women who interacted with the counter-stereotypic avatar (e.g., female and “cool”) reported higher self-efficacy and interest in engineering (Plant et al. 2009; Rosenberg-Kima et al. 2008). We suspect that this interaction task led women to view the prototypical engineer in less stereotypic terms and in ways more similar to themselves. Thus, one approach to combat gender differences in perceptions of CS&E prototypes might be to give young men and women direct experience with counter-stereotypic exemplars in these fields. Such an intervention strategy should lead to less extreme perceptions of prototypical individuals in CS&E and, especially for women, may increase interest in these fields.

### Conclusions

There exists a large and pervasive gender gap in computer science and engineering careers in the United States (U.S. Department of Labor 2016). Our work offers important new insights into gender differences in people’s perception of CS&E prototypes that undermine women’s interest in these important STEM fields. As we have suggested, we think the current work provides exciting avenues for future interventions designed to increase women’s representation in these vital fields by encouraging more accurate, less stereotype-based perceptions of prototypical individuals in CS&E. Indeed, such intervention work has the potential to increase women’s participation in CS&E in two ways. First, we would expect a direct effect of interventions designed to promote more inclusive prototype perceptions with respect to intervention recipients’ interest in CS&E. More importantly, to the degree that this and other effective strategies for increasing representation of women in STEM fields are effective (e.g., Miyake et al. 2010; Stout et al. 2011), even women who are not direct recipients of interventions are likely to notice the resulting increased diversity within STEM. Thus, the best way to change women’s perceptions of CS&E prototypes may be to truly improve representation of women in these fields and to make sure that this improved diversity is broadly visible, thereby improving men’s and women’s perceptions of individuals in these important STEM fields.

## Electronic supplementary material

ESM 1 (DOCX 27 kb)
