# Supplementary material for: Do Gender Differences in Perceived Prototypical Computer Scientists and Engineers Contribute to Gender Gaps in Computer Science and Engineering?
Source: Sex Roles. 2017 Apr 7;78(1):40–51. doi: 10.1007/s11199-017-0763-x (PMC5756563; doi:10.1007/s11199-017-0763-x)
Supplement: Supplementary file 1 — (DOCX 27 kb) [file 11199_2017_763_MOESM1_ESM.docx]

Online Supplement for Ehrlinger, J., Plant, E. A., Hartwig, M. K., Vossen, J. J., Columb, C. J., & Brewer, L. E. (2017). Do gender differences in perceived prototypical computer scientists and engineers contribute to gender gaps in computer science and engineering? *Sex Roles*. Joyce Ehrlinger, Washington State University. Email: [Ehrlinger@wsu.edu](mailto:Ehrlinger@wsu.edu)

| Table 1s: Factors for Ratings of the Self and the CS Prototype (Study 1) - Women | | | | | | | | | | | |
| --- | --- | --- | --- | --- | --- | --- | --- | --- | --- | --- | --- |
|  | CS Prototype Rating  Factors | | | |  | Self-Rating  Factors | | | | |  |
| Item | 1 | 2 | 3 | 4 | Item | 1 | 2 | 3 | 4 | 5 |  |
| Cynical | .41 |  | .66 |  | Cynical |  |  |  | .56 |  |  |
| Artistic | .79 |  |  |  | Artistic | .91 |  |  |  |  |  |
| Creative | .75 |  |  |  | Creative | .84 |  |  |  |  |  |
| Energetic | .81 |  |  |  | Energetic |  | -.59 |  |  | .52 |  |
| Studious | -.56 |  |  |  | Studious |  |  |  |  | .76 |  |
| Intelligent |  | .64 |  |  | Intelligent |  |  |  |  | .57 |  |
| Logical |  | .85 |  |  | Logical |  | .51 | .64 |  |  |  |
| Mathematical |  | .90 |  |  | Mathematical |  | .56 |  |  |  |  |
| Introverted |  |  | .63 |  | Introverted |  | .80 |  |  |  |  |
| Social |  |  | -.53 |  | Social |  | -.78 |  |  |  |  |
| Clumsy |  |  |  | .73 | Clumsy |  |  | -.88 |  |  |  |
| Insecure |  |  |  | .75 | Insecure |  |  |  | .82 |  |  |
| Athletic |  |  |  |  | Athletic |  |  |  |  | .72 |  |
| % of variance explained | 28.1% | 13.3% | 10.6% | 8.7% |  | 22.2% | 17.2% | 10.7% | 9.6% | 8.1% |  |
| *Note.* Results from direct oblimin rotations performed separately on prototype and self-ratings. All loadings greater than .40 are shown. | | | | | | | | | | | |

| Table 2s: Factors for Ratings of the Self and the CS Prototype (Study 1) - Men | | | | | | | | | | | |
| --- | --- | --- | --- | --- | --- | --- | --- | --- | --- | --- | --- |
|  | CS Prototype Rating  Factors | | | |  | Self-Rating  Factors | | | | |  |
| Item | 1 | 2 | 3 | 4 | Item | 1 | 2 | 3 | 4 | 5 |  |
| Athletic | .86 |  |  |  | Athletic | .72 |  |  |  |  |  |
| Cynical | -.68 |  |  |  | Cynical |  | -.87 |  |  |  |  |
| Artistic | .62 |  |  |  | Artistic |  |  |  | -.77 |  |  |
| Creative | .52 |  | .61 |  | Creative |  |  |  | -.88 |  |  |
| Energetic | .71 |  |  |  | Energetic | .66 |  | .45 |  |  |  |
| Insecure | -.54 |  |  | .40 | Insecure |  |  |  |  | .80 |  |
| Mathematical | -.46 |  | .56 | .45 | Mathematical |  |  |  |  | .81 |  |
| Studious | -.64 |  |  |  | Studious |  |  | .82 |  |  |  |
| Clumsy |  | .53 | .51 |  | Clumsy | -.54 | .42 | .45 |  |  |  |
| Introverted |  | .88 |  |  | Introverted | -.66 |  |  |  |  |  |
| Social |  | -.88 |  |  | Social | .77 |  |  |  |  |  |
| Intelligent |  |  | .78 |  | Intelligent |  | -.51 | .58 |  |  |  |
| Logical |  |  |  | .88 | Logical |  | -.70 |  |  |  |  |
| % of variance explained | 28.7% | 14.8% | 11.7% | 9.6% |  | 23.9% | 16.3% | 13.2% | 11.6% | 8.8% |  |
| *Note.* Results from direct oblimin rotations performed separately on prototype and self-ratings. All loadings greater than .40 are shown. | | | | | | | | | | | |

Table 3s; Correlations between age and the dependent measures in Study 2.

|  | Correlations with Age | |
| --- | --- | --- |
| Dependent measures | *r* | *p* |
| Self-Ratings | -.12 | .12 |
| E Prototype Ratings | .02 | .79 |
| Perceived Similarity | .08 | .29 |
| Interest in Engineering | -.06 | .44 |
| Exposure to Engineering | -.01 | .89 |

*Note.* All correlations with age were NS.

| Table 4s: Factors for Ratings of the Self and the Engineering Prototype (Study 2) - Women | | | | | | | | |  |
| --- | --- | --- | --- | --- | --- | --- | --- | --- | --- |
|  | Engineering Prototype Rating Factors | | |  | Self-Rating  Factors | | | | |
| Item | 1 | 2 | 3 | Item | 1 | 2 | 3 | 4 | |
| Introverted | .55 |  | .47 | Introverted | .78 |  |  |  | |
| Shy | .83 |  |  | Shy | .86 |  |  |  | |
| Social | -.89 |  |  | Social | -.87 |  |  |  | |
| Athletic | -.83 |  |  | Athletic |  |  | -.71 |  | |
| Intelligent |  | .67 |  | Intelligent |  | .78 |  |  | |
| Mathematical |  | .80 |  | Mathematical |  | .51 |  |  | |
| Logical |  | .78 |  | Logical |  | .77 |  |  | |
| Cynical |  |  | .88 | Cynical |  |  |  | .88 | |
| Clumsy |  |  |  | Clumsy |  |  | .80 |  | |
| % of variance explained | 31.9% | 19.1% | 11.3% |  | 26.2% | 17.7% | 13.5% | 11.3% | |
| *Note.* Results from direct oblimin rotations performed separately on prototype and self-ratings. All loadings greater than .40 are shown. | | | | | | | | | |

| Table 5s: Factors for Ratings of the Self and the Engineering Prototype (Study 2) - Men | | | | | | | |  |
| --- | --- | --- | --- | --- | --- | --- | --- | --- |
|  | Engineering Prototype Rating Factors | | |  | Self-Rating  Factors | | | |
| Item | 1 | 2 | 3 | Item | 1 | 2 | 3 | |
| Intelligent | .92 |  |  | Intelligent |  |  | -.62 | |
| Mathematical | .87 |  |  | Mathematical |  | .54 |  | |
| Clumsy | -.41 | -.72 |  | Clumsy |  |  | -.84 | |
| Logical | .68 |  | .47 | Logical |  | .70 |  | |
| Shy |  | -.68 |  | Shy | .78 |  |  | |
| Social |  | .85 |  | Social | -.66 |  |  | |
| Athletic |  | .79 |  | Athletic |  | .72 |  | |
| Introverted |  |  | .57 | Introverted | .75 |  |  | |
| Cynical |  |  | .82 | Cynical | .48 |  |  | |
| % of variance explained | 28.9% | 26.4% | 13.3% |  | 24.4% | 19.5% | 11.7% | |
| *Note.* Results from direct oblimin rotations performed separately on prototype and self-ratings. All loadings greater than .40 are shown. | | | | | | | | |

Table 6s. Mean ratings of CS prototypes (Study 1) or Engineering prototypes (Study 2) for each separate trait and whether gender differences in those ratings were significant.

|  | Study 1 (CS) | | |  | Study 2 (Engineering) | | |
| --- | --- | --- | --- | --- | --- | --- | --- |
| Trait/Characteristic | Female | Male | Difference |  | Female | Male | Difference |
| intelligent | 8.30 | 7.89 | 0.42* |  | 8.56 | 8.05 | 0.51* |
| mathematical | 8.43 | 7.93 | 0.51* |  | 8.72 | 8.27 | 0.45* |
| logical | 8.17 | 8.07 | 0.10 |  | 8.27 | 7.73 | 0.54* |
| creative | 5.25 | 5.63 | -0.38 |  | -- | -- | -- |
| artistic | 3.96 | 4.11 | -0.15 |  | -- | -- | -- |
| studious | 7.71 | 6.93 | 0.78* |  | -- | -- | -- |
| cynical | 5.06 | 5.22 | -0.16 |  | 4.75 | 5.18 | -0.44 |
| insecure | 5.22 | 5.37 | -0.15 |  | -- | -- | -- |
| shy | -- | -- | -- |  | 5.22 | 5.18 | 0.04 |
| introverted | 6.80 | 6.19 | 0.61 |  | 5.47 | 5.20 | 0.28 |
| social | 3.30 | 4.37 | -1.07* |  | 4.84 | 4.45 | 0.38 |
| energetic | 4.83 | 4.85 | -0.03 |  | -- | -- | -- |
| athletic | 3.17 | 2.78 | 0.40 |  | 4.45 | 4.02 | 0.43 |
| clumsy | 4.80 | 5.07 | -0.28 |  | 3.48 | 4.33 | -0.84* |

*Note*. Positive differences indicate females gave higher ratings than males did; negative differences indicate females gave lower ratings than males did. **p* < .05
